# Strain Imaging and Ventricular Arrhythmia

**DOI:** 10.3390/diagnostics13101778

**Published:** 2023-05-17

**Authors:** Caroline Løkke Bjerregaard, Kristoffer Grundtvig Skaarup, Mats Christian Højbjerg Lassen, Tor Biering-Sørensen, Flemming Javier Olsen

**Affiliations:** 1 Department of Cardiology, Copenhagen University Hospital–Herlev and Gentofte, 2900 Hellerup, Denmark; bjerregaard.caroline@gmail.com (C.L.B.); kristofferskaarup@hotmail.com (K.G.S.); mcha@live.dk (M.C.H.L.); tor.biering@gmail.com (T.B.-S.); 2 Center for Translational Cardiology and Pragmatic Randomized Trials, Department of Biomedical Sciences, Faculty of Health and Medical Sciences, University of Copenhagen, 2200 Copenhagen, Denmark

**Keywords:** strain, arrhythmia, mechanical dispersion, dyssynchrony, myocardial work

## Abstract

Ventricular arrhythmia is one of the main causes of sudden cardiac death. Hence, identifying patients at risk of ventricular arrhythmias and sudden cardiac death is important but can be challenging. The indication for an implantable cardioverter defibrillator as a primary preventive strategy relies on the left ventricular ejection fraction as a measure of systolic function. However, ejection fraction is flawed by technical constraints and is an indirect measure of systolic function. There has, therefore, been an incentive to identify other markers to optimize the risk prediction of malignant arrhythmias to select proper candidates who could benefit from an implantable cardioverter defibrillator. Speckle-tracking echocardiography allows for a detailed assessment of cardiac mechanics, and strain imaging has repeatedly been shown to be a sensitive technique to identify systolic dysfunction unrecognized by ejection fraction. Several strain measures, including global longitudinal strain, regional strain, and mechanical dispersion, have consequently been proposed as potential markers of ventricular arrhythmias. In this review, we will provide an overview of the potential use of different strain measures in the context of ventricular arrhythmias.

## 1. Introduction

Ventricular arrhythmias (VA) pose a substantial risk for the development of sudden cardiac death (SCD) [1,2]. While VA may develop because of channelopathies, toxicity, or for idiopathic reasons, structural heart disease is a frequent cause of VA [3]. Cardiac imaging, including echocardiography, may help detect structural and functional heart disease to identify patients at risk of VA. In line with this, the estimation of systolic function by left ventricular ejection fraction (LVEF) is used to guide the indication for the implantation of an implantable cardioverter defibrillator (ICD) in heart failure (HF) [3,4]. However, LVEF is limited by its pure volume-based assessment of systolic function and by technical constraints, including poor reproducibility, geometric assumptions, and loading dependency [5,6]. Consequently, LVEF has shown to be limited in terms of predicting VAs, particularly in patients with LVEF >35%, and early prediction of the occurrence of VAs remains a challenge despite being the most common cause of SCD [1,2,3,5,7].

Given the constraints of LVEF and the varying pathophysiological mechanisms that may promote the development of VAs, several studies have evaluated the potential prognostic utility of other biomarkers in the context of VAs. These include electrocardiographic markers as well as findings from cardiac magnetic resonance imaging, computed tomography, radionuclide imaging, and novel echocardiographic techniques [8,9,10,11,12,13,14,15,16,17,18,19,20,21].

Speckle tracking is one of the most promising echocardiographic techniques in this regard. As outlined in Table 1, speckle tracking offers several advantages for the assessment of myocardial function. Strain imaging parameters, in particular global longitudinal strain (GLS), have been shown to be more sensitive markers of cardiovascular risk than LVEF [22,23,24,25,26]. Accordingly, strain imaging could be of value in terms of predicting VAs and may potentially aid in the selection of candidates who would benefit from an ICD.

This review will provide an overview of the potential use of different strain imaging measures as predictors of VA. Although this has been investigated in various patient groups, it has most widely been studied in the settings of ischemic heart disease and HF. This review will therefore focus on the utility of strain measures in these conditions but also highlight other potential disease categories.

## 2. Concept of Myocardial Strain Imaging

Strain imaging was initially introduced as a derivative of tissue Doppler imaging but was made quantifiable through speckle tracking in 2004 by Lysyanski et al. [27,28]. The method of speckle tracking takes advantage of the presence of natural acoustical markers within the myocardium that can be tracked throughout the cardiac cycle [29]. Speckle tracking is therefore possible in multiple projections, typically measured in either apical or short-axis views, and allows for the quantification of longitudinal, circumferential, and radial strain, as well as twist and torsion [30]. Key practical points to consider include proper visualization of myocardial tissue, adequate temporal resolution (~60–90 fps), and careful consideration of ECG trigger points, but more details are available in published practical guidelines [31]. The longitudinal strain has been most widely assessed because of its higher feasibility and reproducibility [5,6] but also because longitudinal fibers located in the subendocardial layer are most susceptible to ischemia [32,33]. Accordingly, most available evidence relies on findings from longitudinal strain. The assessment of longitudinal strain can provide widespread insight into cardiac mechanics, including quantification of GLS, regional strain, mechanical dispersion (MD), and, most recently, myocardial work as potential markers of cardiovascular outcomes, including the risk of arrhythmias.

## 3. Global Longitudinal Strain

### 3.1. Value of GLS

As indicated by the name, GLS represents a global measure of LV myocardial tissue deformation acquired from the three main apical projections. Since the introduction of myocardial speckle tracking, several studies have emerged to suggest that GLS can detect LV systolic dysfunction at an earlier point than LVEF [34,35]. Several technical and pathophysiological aspects contribute to why that is. As mentioned above, GLS evaluates myocardial deformation and, thereby, contractile tissue function, whereas LVEF is a volume-based measurement and, thereby, an indirect surrogate of systolic function. In addition, GLS specifically evaluates longitudinal fiber function, which is more sensitive to ischemia since coronary perfusion extends from the subendocardium and outwards [36,37,38]. Finally, GLS has been shown to correlate closely with the neurohumoral response, infarct size, and myocardial fibrosis [39,40,41]. 

The primary impediment that has prevented GLS from earlier widespread clinical implementation has been its vendor dependency [42,43]. However, collaborations have already been created to mitigate this issue [44]. In addition, data from several studies proposing normal values for GLS are now available, further benchmarking GLS for clinical practice. A large-scale meta-analysis of ~2600 subjects reported that normal values varied from −15.9 to −22.1% with a mean value of −19.7% [45], and a rule of thumb has been that −16 to −18% is considered borderline abnormal, whereas a lower threshold of −16% was considered abnormal [46]. This lower threshold was recently validated in ~2000 healthy participants from a general population study (the Copenhagen City Heart study), which found −15.8% to be the lower limit of normality for GLS [47]. Finally, GLS has consistently shown excellent reproducibility, and by direct comparison, the reproducibility of GLS was substantially better than LVEF, regardless of the operator’s level of expertise [48]. These efforts have promoted the dissemination of GLS into various guidelines and recommendations [49,50].

### 3.2. GLS as a Marker of Ventricular Arrhythmia

**Figure 1:**
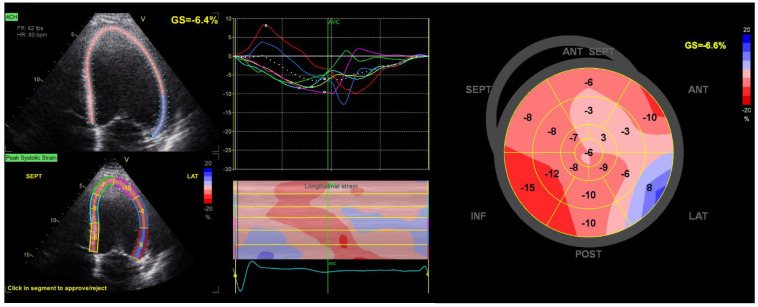
Global and regional strain. The figure shows an example of strain analysis in a patient with ischemic cardiomyopathy and ICD who experienced appropriate therapy after implantation. On the left side, speckle tracking of the left ventricle in the apical 4–chamber view is shown. In the middle panel, strain profiles for each segment are shown (colored curves), and the global value for this projection is shown as the white dotted profile. On the right, a corresponding bulls–eye plot of regional strain values is shown, and the global strain value is denoted as ‘GS’ in the upper right-hand corner. Overall, this patient had markedly reduced global longitudinal strain and widespread regional abnormalities in longitudinal strain, most notable for the basal lateral wall segment, showing positive strain values indicating paradoxical motion.

As an extension to simply recognizing systolic dysfunction, several observational studies have also shown that GLS can predict cardiovascular outcomes even in patients with preserved LVEF [34,51,52]. Such findings also extend into the context of the prediction of VA. Figure 1 is a representative example of GLS measured in a patient at risk of VA. Table 2 provides an outline of studies examining GLS in relation to VAs across various cardiac disorders. Even though GLS has shown potential for predicting VAs, it should be noted that heterogeneity exists concerning endpoint definition, rhythm monitoring, and length of follow-up.

For the selection of candidates for ICD implantation as a primary preventive strategy, current guidelines rely heavily on an LVEF threshold of <35% [3], despite studies showing that VA and SCD risk stratification by LVEF may be suboptimal [53,54,55]. Ersbøll et al. reported on the value of GLS for predicting VA in the acute setting of myocardial infarction (MI) with echocardiograms performed within 48 h of admission (n = 988 with 34 events during 29.7 months of follow-up) in a prospective study. The authors found that GLS was significantly reduced among patients who developed VAs compared to those who did not (9.9% vs. 13.9%, *p* < 0.001) and that GLS was an independent predictor of VAs after adjusting for clinical, electrocardiographic, and echocardiographic parameters. In addition, GLS improved risk prediction beyond LVEF and Killip class. Finally, GLS was found to be an independent predictor of VAs even in patients with LVEF above 35%, further emphasizing the potential added value of GLS [56]. However, landmark analysis from the study suggested that GLS, as opposed to MD, was significantly associated with VAs in the short term (within 90 days) and highlighted the fact that there could be a diminishing return on its predictive value with increasing length of time from the index infarction as compared to MD. Indeed, in a multicenter, prospective study of 569 patients (15 events during 30 months of follow-up), with echocardiograms performed >40 days after the MI, Haugaa et al. found both GLS and MD to be univariable predictors of VAs; however, when both parameters were included in the same regression model, only MD remained significantly associated with VAs [57]. Central limitations to both these studies, as well as others, have been the use of admission with VAs rather than systematic rhythm monitoring for endpoint ascertainment as well as the few numbers of events. It is also worth noting that in an antecedent study by Haugaa et al. on post-MI patients with ICDs (n: 85 with 38 events during 2.3 years of follow-up), only MD and not GLS was found to be an independent predictor of appropriate ICD therapy [58]. It should, however, be noted that even though this was a prospective study, the echocardiograms were performed at various time points from ICD implantation, and the median time from the MI to the study’s baseline was about 6 years. Accordingly, the lack of independent predictive value for GLS could be related to the long time that elapsed from MI to echocardiogram. 

As can be appreciated above and from Table 2, some inconsistencies appear concerning GLS’ predictive value, particularly in ischemic heart disease. To that end, a meta-analysis based on undifferentiated populations reported that GLS was not associated with VA events (2076, events: 147) [59]. However, it should be noted that in another meta-analysis based on 984 patients with non-ischemic cardiomyopathy (231 events), GLS was found to be significantly associated with VAs [60]. It is also worthwhile to mention that in a MADIT-CRT substudy, representing the largest study that has examined GLS’ value with continuous rhythm monitoring and adjudicated VA events (n = 1064, events: 254), GLS was also found to be associated with VAs [61]. Compared to meta-analyses, which extrapolate results based on events that may differ markedly in terms of rhythm monitoring and definition of VA endpoints, large-scale studies such as MADIT-CRT provide findings from a more homogenous design. 

In addition to the above-mentioned studies, several other studies have also reported on the potential of GLS for predicting VAs in MI patients [62,63,64]. A brief outline of these studies is provided in Table 2; however, the findings are less easily interpreted since the studies were either retrospective or included VAs in a composite outcome with HF or mortality.

**Table 2:** Global longitudinal strain and ventricular arrhythmia.

Study	Year	Design	Sample Size	Arrhythmia Outcome	Arrhythmia Monitoring	No. of Events	Follow-Up	Key Strain Findings
Myocardial infarction								
Haugaa et al. [58]	2010	Prosp.	85	Appropriate ICD therapy	ICD monitoring	38	2.3 (range: 0.6–5.5) years	MD but not GLS was an independent predictor of appropriate ICD therapy.
Haugaa et al. [57]	2013	Prosp.	569	Composite: Sustained VT VF SCD	Not specified	15	30 (IQR: 18) months	GLS was a univariate predictor of VA but not an independent predictor of VA when adjusted for MD. MD was an independent predictor of VA.
Ersbøll et al. [56]	2013	Prosp.	988	Composite: VA Appropriate ICD therapy Definite/suspected SCD	Admission with documented VA ICD monitoring in subgroup SCD based on hospital and prehospital records.	34	29.7 (IQR: 23.5–32.7) months	GLS and MD were independent predictors of VA. In patients with LVEF < 35%, both GLS and MD were independent predictors of VAs, but only GLS was an independent predictor of VAs in patients with LVEF > 35%.
Sjøli et al. [62]	2011	Prosp.	77	Composite: Cardiac death Reinfarction Hospitalization for HF UAP Life-threatening arrhythmia	Not specified	17	3.29 ± 1.59 (range: 0–5.22) years	GLS measured in both the acute phase and after 10 days was an independent predictor of the composite outcome.
Nguyen et al. [63]	2015	Not specified	467	VT	Documented on 24 h ambulatory ECG monitoring during hospitalizationEP study	51	Median: 25 (range: 6–43) months	In multivariate analysis, MD was significantly associated with VT, and GLS was borderline significantly associated with VT.
Choi et al. [64]	2022	Retrosp.	545	Composite: All-cause death Rehospitalization for acute HF VA	National database and electrical medical records	55	Median: 49.5 months	Reduced 3D and 2D GLS were both independently associated with the composite outcome.
Leong et al. [65]	2015	Retrosp.	206	Appropriate ICD therapy	ICD monitoring	75	Median: 24 (IQR: 7.8–24) months	GLS and MD were independently associated with VT.
Structural heart disease								
Guerra et al. [66]	2020	Prosp.	203	Any VA detected by ICD	ICD monitoring	74	817 (IQR: 440–1105) days	GLS Ws an independent predictor of the first VA episode but not recurrent episodes. MD was not associated with VAs.
Heart failure with reduced ejection fraction								
Nikoo et al. [67]	2020	Prosp.	70	Appropriate ICD therapy	ICD monitoring	30	1.8 ± 0.6 (1–3) years	Reduced GLS was a predictor of VAs. Better diagnostic performance than LVEF. MD was not reported.
Hasselberg et al. [68]	2016	Prosp.	170	Composite: VT VF SCA Appropriate ATP Appropriate defibrillator shock therapy	CRT-D monitoring	18	1.9 ± 0.3 years	GLS and MD at baseline were not independent predictors of the VA endpoint. MD at 6 months was an independent predictor of the VA endpoint.
Mornoş et al. [69]	2017	Prosp.	340	Composite: VT VF SCD	Hospital documentationDeath certificate	48	36 ± 9 months	GLS, MD, and the ratio of GLS to MD (GLS/MD) were univariate predictors of VAs, but only GLS/MD was an independent predictor of VAs.
Matsuzoe et al. [70]	2016	Retrosp.	72	Appropriate ICD therapy	ICD monitoring	34	17 (IQR: 0.2–72.5) months	GLS and MD were not independently associated with the VA endpoint. Only LV dyssynergy (SD of peak strain) was independently associated with the VA endpoint.
Biering-Sørensen et al. [61]	2017	RCT substudy	1064	Appropriate ICD/CRT-D therapy	ICD/CRT-D monitoring Adjudicated events	254	2.9 (IQR: 2.0–3.7) years	GLS and all regional (anterior and inferior) strain were associated with VT/VF, whereas MD was not.
Bax et al. [71]	2017	RCT substudy	755	Composite: Appropriate ICD/CRT-D therapy Arrhythmic death Atrial tachyarrhythmias	ICD/CRT-D monitoring Adjudicated events	72	19.4 months	GLS was not independently associated with the arrhythmic endpoint. MD was not investigated.
Biering-Sørensen et al. [72]	2016	Retrosp.	151	Composite: CVD Appropriate ICD therapy	ICD monitoring CVD from the national cause of death registry	40	2.3 (IQR: 1.5–3.1) years	Neither MD nor GLS was associated with VAs.
Winsløw et al. [73]	2023	RCT substudy	401	Composite: SCD Appropriate ICD therapy Admission with sustained ventricular arrhythmia Resuscitated cardiac arrest	ICD monitoring ECG Hospital/source documentation Adjudicated events	52	4.0 (IQR:2.8–5) years	Neither GLS nor LVEF was associated with the VA endpoint. Only inferior strain was independently associated with the VA endpoint.
Non-ischemic dilated cardiomyopathy								
Haugaa et al. [74]	2012	Prosp.	94	Composite: Appropriate ICD therapy Sustained VT Cardiac arrest Cardiac syncope	Not specified	12	22 (Range:1–46) months	Both GLS and MD were independent predictors of the VA endpoint.
Melichova et al. [75]	2021	Prosp.	290	Composite: SCD Shock from ICD Sustained VT	Medical records (ICD therapy, ECG, Holter, aborted cardiac arrest) Cause of death registry	32	22 ± 12 months	Both GLS and MD were independent predictors of the VA endpoint.
Negishi et al. [76]	2016	Retrosp.	124	Appropriate ICD therapy	ICD monitoring	36	3.8 (IQR: 2.2–6.0) years	GLS, but not MD, was an independent predictor of VAs.
Hypertrophic cardiomyopathy								
Haland et al. [77]	2016	Prosp.	150 HCM	Composite: Sustained and non-sustained VT Previous aborted cardiac arrest	24–48 h Holter monitoring ICD monitoring	37	Not specified	GLS and MD were univariate predictors of the VA endpoint, but only MD was an independent predictor.
Candan et al. [78]	2017	Prosp.	63	Appropriate ICD therapy	ICD monitoring	17	3 years (21.5 ± 6.9 months)	GLS and MD were independent predictors of VAs.
Debonnaire et al. [79]	2014	Retrosp.	92	Appropriate ICD therapy	ICD monitoring	21	4.7 (2.2–8.2) years	GLS was independently associated with VAs. MD was not investigated.
Candan et al. [80]	2019	Prosp.	59	Non-sustained VT	24–72 h Holter monitoring	17	N/A	LV Twist and GLS were independent predictors for non-sustained VT. MD was not investigated.
Popa-Fotea et al. [81]	2020	Prosp.	47	Non-sustained VT	24 h Holter monitoring	16	N/A	GLS, RV and LV MD were univariate predictors of non-sustained VT, but only RV and LV MD were independent predictors of non-sustained VT.
Hiemstra et al. [82]	2017	Prosp.	427	Composite: Aborted SCD Appropriate ICD therapy	Medical chart review Contact with general practitioner	53	6.7 (IQR: 3.3–10.0) years	GLS was independently associated with the VA endpoint. MD was not investigated.
Jalanko et al. [83]	2016	Prosp.	31	Non-sustained VT	24 h Holter monitoring	11	N/A	Both GLS and MD were associated with non-sustained VT in univariate analysis, but only MD was independently associated with non-sustained VT.
Chagas cardiomyopathy								
Barros et al. [84]	2016	Retrosp., case-control study	62	Clinically indicated implantation of ICD.	N/A	28	N/A	MD and GLS were more abnormal in the group with ICD, and both were independent markers of previous events precipitating ICD.
Azevedo et al. [85]	2021	Prosp.	77	Composite: VES Non-sustained VT	24 h Holter	Not specified	N/A	Both GLS and MD were associated with non-sustained VT in univariate analysis, but only MD was independently associated with non-sustained VT, paired VES, and VES in bigeminy.
Long QT syndrome								
Haugaa et al. [86]	2010	Prosp.	101 LQTS 35 healthy individuals	History of either: Documented arrhythmia Syncope Cardiac arrest	N/A	48	N/A	LQTS patients with a history of arrhythmia had higher MD but similar GLS compared to those without arrhythmia.
Lamin A/C mutation								
Haugaa et al. [87]	2015	Prosp.	33	Composite: Non-sustained VT VT VF	Not specified	11	Not specified	Patients with any ventricular arrhythmia had higher MD but similar GLS compared to those without ventricular arrhythmia.
Tetralogy of Fallot								
Diller et al. [88]	2012	Retrosp.	413	Composite: SCD Sustained VTResuscitated SCD Appropriate ICD discharge	ICD monitoring	19	2.9 (IQR:1.4–4.4) years	GLS was an independent predictor of the VA endpoint. MD was not investigated.
Van Grootel et al. [89]	2019	Prosp.	151 ToF	Composite: Death HF Reintervention Hospitalization for cardiac reasons Symptomatic ventricular and supraventricular arrhythmias	Regularly checked at an outpatient clinic	62	71.5 (IQR: 64–75.3) months	GLS, RV strain, and apical rotation were univariate predictors of the composite outcome. Only apical rotation was independently associated with the composite outcome. MD was not investigated.
Cardiac amyloidosis								
Hamon et al. [90]	2016	Prosp.	45	Appropriate ICD therapy	ICD monitoring	12	17 ± 13.7 months	GLS was not associated with VAs. MD was not investigated.
Brugada syndrome								
Scheirlynck et al. [91]	2020	Case-control study	175 BrS	History of either: VT VF Aborted cardiac arrest	Medical records	19	N/A	Patients with a history of VAs or aborted cardiac arrest had higher MD than, but similar GLS to, those who had not had VAs or aborted cardiac arrest.
Elite Athletes								
Lie et al. [92]	2021	Cross-sectional study	43 athletes with VT and 30 healthy athletes	Composite of life-threatening VAs: VF Sustained VT Aborted cardiac arrest Appropriate ICD therapy	24 h Holter monitoring ECG Telemetry ILR monitoring Intracardiac device monitoring	23	N/A	MD was higher and GLS was lower in VA patients. Only MD was independently associated with life-threatening VAs.
Arrhythmogenic cardiomyopathy								
Lie et al. [93]	2018	Prosp.	117	VT Cardiac arrest Appropriate ICD shock	ECG Holter monitoring ICD monitoring	18	2.0 (IQR:0.5–3.5) years	Patients with VAs had reduced LV and RV strain and higher LV and RV MD. RV strain and LV MD were independently associated with VAs.
Lie et al. [94]	2021	LCS	168	Composite: Aborted cardiac arrest Sustained VT Appropriate ICD shock	Not specified	54	1.3 (IQR: 0.4–3.5) years	LV GLS was independently associated with VAs. MD was not reported.
Sarvari et al. [95]	2011	Prosp. Case-control study	42 symptomatic 27 asymptomatic 30 healthy	History of either: VT VF	N/A	42	N/A	Patients with a history of VAs had lower LV and RV strain and higher LV and RV MD. Only RV MD was independently associated with a history of VAs.
Kirkels et al. [96]	2021	Retrosp.	160	History of either: Sustained VT Appropriate ICD therapy Aborted cardiac arrest	N/A	47	N/A	Patients with a history of VAs had reduced LV GLS and RV strain and higher RV MD than those without VA history. RV MD was independently associated with VAs.
Mitral valve prolapse								
Ermakov et al. [97]	2019	Retrosp.	59 MVP	History of: Ventricular couplets Ventricular bigeminy Non-sustained VT VT ICD for aborted cardiac arrest	N/A	32	N/A	MD higher but similar GLS in patients with a history of VA compared to those without arrhythmia. MD was independently associated with a history of VA.
Unexplained syncope								
Falsing et al. [98]	2021	Retrosp.	288	VT	ILR monitoring	36	2.9 (IQR:1.3–3.5) years	GLS was independently associated with VT. MD was not associated with VT.
Idiopathic ventricular fibrillation								
Groeneveld et al. [99]	2021	Retrosp. Case-control study	47 IVF 47 healthy individuals	VF	N/A	N/A	N/A	IVF patients had lower GLS, higher MD, and higher post-systolic index than matched controls. No adjusted analyses were performed.
Acute myocarditis								
Pruitt et al. [100]	2021	Retrosp.	66	Composite: VT VF SVT High-grade or complete heart block Any arrhythmia requiring antiarrhythmic medication	Medical records	23	During hospitalization	GLS was independently associated with the composite arrhythmia outcome.

Abbreviations: Prosp., prospective; Retrosp., retrospective; HCM, hypertrophic cardiomyopathy; LQTS, long QT syndrome; MVP, mitral valve prolapse; Brs, Brugada syndrome; VT, ventricular tachycardia; IVF, idiopathic ventricular fibrillation; ICD, implantable cardioverter-defibrillator; SCD, sudden cardiac death; VF, ventricular fibrillation; SCA, sudden cardiac arrest; CRT, cardiac resynchronization therapy; VES; ventricular extrasystoles; HF, heart failure; VA, ventricular arrhythmia; ECG, electrocardiogram; GLS, global longitudinal strain; MD, mechanical dispersion; LVEF, left ventricular ejection fraction; LV, left ventricle; RVMD, right ventricular mechanical dispersion; RV, right ventricle; MD, mechanical dispersion; LBBB, left bundle branch block.

## 4. Regional Strain

### 4.1. Is Regional Strain Worthwhile to Consider?

Since myocardial strain imaging can be performed from multiple projections, the assessment of tissue deformation within specific regions of the LV is feasible. Cardiac MRI studies have shown that radial, circumferential, and longitudinal strain deteriorate in a stepwise fashion from non-infarcted areas to peri-infarct regions and infarcted regions after a MI [101]. Since GLS reflects global LV function, regional LV strain could be of potential value to detect specific areas of systolic dysfunction. In fact, in a subset of participants in the VALIANT study, the authors showed that regional strain was impaired even in segments that were visually estimated to be normokinetic and that a higher number of regions with abnormal strain posed a higher risk of death [102]. It should be noted, though, that studies relating regional strain to outcome have been inconsistent. Conceptually, lower regional strain in infarcted areas was thought to pose an increased risk of outcomes such as HF and all-cause death. However, a study by Biering-Sørensen et al. found that reduced regional strain outside the culprit perfusion area was a more important aspect to consider, as this would indicate limited compensatory reserve after an MI [103]. In addition to the above-mentioned considerations, the regional strain has also been shown to be associated with other outcomes after MI, including LV thrombus formation [104]. 

It is, however, worthwhile to note that compared to GLS, regional strain is even more heterogenous across different vendor solutions and exhibits poorer reproducibility [105,106]. A representative example of regional strain in a patient with ischemic cardiomyopathy is shown in Figure 1.

### 4.2. Regional Strain and Risk of Ventricular Arrhythmia

Several underlying mechanisms could indicate a potential for regional strain as a marker of VA risk, one being the ability to identify regions within an infarct zone or in the peri-infarct zone that may contribute to arrhythmogenic potential. This relies on the fact that in patients with MI, areas of the infarcted zone commonly consist of fibrotic tissue and represent an anatomical and physiological substrate for malignant arrhythmias [107,108]. Meanwhile, the peri-infarct zone comprises heterogeneous areas with an intermediate degree of non-transmural fibrosis with potential for conduction delay, unidirectional conduction block, and electrical dispersion, thereby creating re-entry substrates that could result in VAs [109]. Indeed, several cardiac MRI studies have demonstrated an ability to anatomically identify myocardial scar tissue, characterize the peri-infarct zone, and quantify the function and extent of the dysfunctional myocardium to predict mortality and the occurrence of VAs [110,111,112,113].

In general, studies on echocardiographically assessed regional strain in the context of VAs are sparse compared to GLS and MD. Bertini et al. investigated 134 patients with chronic ischemic cardiomyopathy scheduled for a clinically indicated electrophysiological (EP) study who had an echocardiogram performed within 24 h prior to the EP study. They found that peak longitudinal systolic strain at the peri-infarct zone was independently associated with inducible VT, whereas peak longitudinal systolic strain values in the infarct and remote zones were not associated with inducibility [114]. By extension, in a prospective study of 424 patients with ischemic cardiomyopathy and prophylactic ICD, Ng et al. investigated the potential of regional strain for predicting appropriate ICD therapy as a secondary outcome. During 24.2 months of follow-up, 95 patients received appropriate ICD therapy. The authors found that regional strain in the peri-infarct zone was independently associated with the occurrence of appropriate ICD therapy [115]. In a related study, Hoogslag et al. investigated the utility of regional strain for predicting a composite outcome of appropriate ICD therapy or cardiac mortality in 79 patients with MI by performing echocardiography at baseline and after 3 months. Interestingly, no difference in regional strain was detected in the infarct, peri-infarct, or remote zone at baseline between patients who developed the outcome and those who did not. However, at the 3-month echocardiogram, it became evident that strain in the peri-infarct zone was reduced in those who developed the outcome, and reduced peri-infarct zone strain was independently associated with the outcome [116]. The findings were extended in a study of 467 patients. During 25 months of follow-up, 51 patients had documentation of VA either on 24 h ECG monitoring, monitoring during hospitalization, or through an EP study. Interestingly, both longitudinal and circumferential strain yielded high AUCs for recognizing VAs, but only abnormal circumferential per-infarct strain was independently associated with VAs along with MD [63]. This may be ascribed to the fact that more extensive MIs would not be limited to subendocardial ischemia and thereby impairment of solely longitudinal function but also reductions in circumferential strain. It is, however, important to keep in mind that none of the above-mentioned studies specifically ascertained where the infarct was located based on late gadolinium enhancement but rather defined the infarct zone based on either regional strain values or wall motion score index.

In the setting of HF, a substudy of the MADIT-CRT trial represents the largest study to report on the potential of regional strain for predicting VAs. Based on 1064 patients with 254 events, Biering-Sørensen et al. reported that longitudinal strain in the inferior and posterior segments of the LV wall was significantly associated with the development of VT/VF and that longitudinal strain obtained from the inferior wall provided prognostic information beyond clinical and echocardiographic parameters for VT/VF [61]. These findings have recently been replicated in a substudy of 401 patients from the DANISH trial with 52 events, also demonstrating an association between inferior wall strain and VAs [73]. The proposed underlying mechanisms for these findings included a differential distribution of parasympathetic and sympathetic innervation to the LV and differential wall stress owing to different radii of curvature across the LV [117,118,119,120,121].

## 5. Mechanical Dyssynchrony

Mechanical dyssynchrony is a term used to describe myocardial contraction inhomogeneity within the LV. Echocardiographic measures of mechanical dyssynchrony have been a point of focus for several decades as a means to identify responders to cardiac resynchronization therapy and predict VA in specific patient groups [57,122,123,124]. The aspect of using dyssynchrony measures in cardiac resynchronization therapy lies beyond the scope of this review, which focuses on the prediction of VA. For that purpose, the most widely investigated dyssynchrony measure has been MD.

### 5.1. Mechanical Dispersion Fundamentals

**Figure 2:**
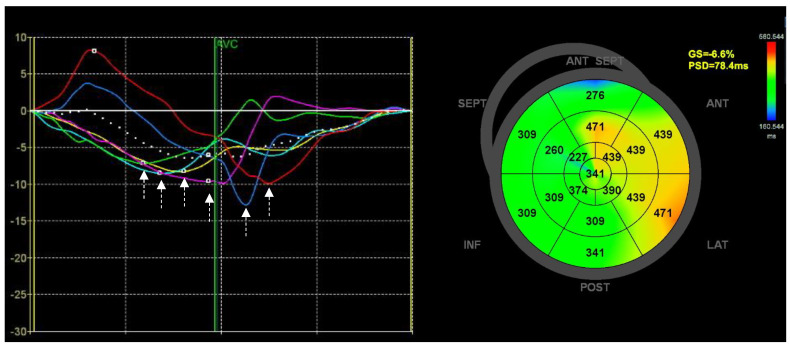
Mechanical dispersion. The figure shows an example of strain analysis, for assessing mechanical dispersion, in a patient with ischemic cardiomyopathy and ICD who experienced appropriate therapy after implantation. On the left, segmental strain profiles from the apical 4–chamber view are shown, with white arrows highlighting a heterogenous timing of peak strain values for the segments (each colored curve represents a single segment). On the right, a bulls–eye plot of time–to–peak strain is shown for all segments, and mechanical dispersion is provided as the peak systolic dispersion (PSD), calculated as the standard deviation of the time–to–peak of the segmental strain.

MD is defined as the standard deviation of the time to peak of the longitudinal strain, typically in a 16-segment model, although this has varied across studies. As for GLS, MD has been reported to have excellent reproducibility with intra- and interobserver ICCs of 0.95 and 0.94, respectively [125]. Figure 2 depicts the heterogeneous contraction of the LV as identified by MD in a patient with ischemic cardiomyopathy.

The presence of LV fibrosis can result in a heterogeneous contraction pattern since fibrosis leads to electrical dispersion, influencing both activation time and refractoriness. This has been noted as the primary underlying mechanism for which MD may be a marker of elevated VA risk [125,126] since MD has been shown to correlate with fibrosis as assessed by LGE [127,128].

Even though MD has been extensively studied, primarily in the context of VA prediction, studies looking at normal reference values based on healthy individuals are sparse. A single study by Rodrígues-Zenella et al. sought to define reference values. Based on 334 healthy volunteers (Caucasian, median age 54 (range: 18–79) years, and 54% women), the authors found an overall normal value of 34 ± 10 ms with an upper limit of normality of 56 ms. Of note, they did not find that MD differed between men and women, but it did increase with age. In addition to proposing reference values, the authors evaluated clinical and echocardiographic correlates and found age, GLS, and E/e’ to be independently correlated with MD [125] in a larger, general population study not restricted to healthy individuals. Aagaard et al. similarly investigated which clinical and echocardiographic parameters correlated to MD, although this study population was not restricted to healthy individuals. Based on 2529 participants, they found coronary artery disease, hypertension, GLS, e’, and LVEF to be significantly associated with higher MD [129].

### 5.2. Mechanical Dispersion and Ventricular Arrhythmias

Similar to GLS, the association between MD and VA has been studied extensively, mostly within HF and coronary artery disease [58,68,69,70,130,131], but also in congenital heart disease [89], long QT syndrome [86,132], and arrhythmogenic cardiomyopathy [93]. An outline of studies is provided in Table 3, and key studies in MI patients have been addressed in the previous section regarding GLS.

Two systematic reviews have assessed the potential of MD for predicting VAs. Recently, in a meta-analysis, Harapoz et al. reported on the potential of MD in patients with non-ischemic cardiomyopathy (n: 346 with 107 events), by which no significant association between MD and VAs was found [60]. However, it should be noted that the authors highlighted that the analysis was limited by the few and relatively small studies, which also precluded an assessment of publication bias. However, it does raise the question as to whether MD is as suitable a predictor of VAs in non-ischemic cardiomyopathy as compared to ischemic cardiomyopathy, which differs in terms of the underlying pathophysiology and distribution of fibrosis. In ischemic cardiomyopathy, a systematic review and meta-analysis by Kawakami et al., which included 12 studies with 3198 patients and 387 arrhythmic events over 17 to 70 months of follow-up, found that MD was higher in patients with VAs, that MD was independently associated with VAs, and that MD was superior to LVEF and GLS for this purpose [59]. It should, however, be noted that the studies included in the meta-analysis were quite heterogenous (I^2^ of 84%), as both patient groups, endpoints, and monitoring differed across the studies. In addition, the timing of the echocardiogram was not considered, which seems to be important in patients with MI, as outlined previously, but also in the context of CRT. Interestingly, Kawakami et al. stated that MD cannot predict VAs in CRT patients since CRT impacts regional timing and the risk of arrhythmias [59]. Indeed, this has been substantiated in substudies from the MADIT-CRT trial [61,133]. As reported by Biering-Sørensen et al., in 1064 patients with continuous rhythm monitoring for a median of 2.9 years, the 254 patients who developed VAs had similar MD as those who remained free of VAs [61]. Before that, Kutyifa et al. reported that baseline MD was not predictive of VAs but noted that CRT patients with LBBB who had improvement in MD at 12 months had a lower risk of VAs [134]. Similar findings have also been noted in a large-scale retrospective study by Van der Bijl al. Based on 1185 patients with 403 events, they did not find that baseline MD was associated with VAs but rather that MD at 6 months was independently associated with VAs [131]. Accordingly, the timing of the echocardiogram seems to be of importance when considering the use of MD for predicting VAs.

**Table 3:** Mechanical dispersion and ventricular arrhythmia.

Study	Year	Design	Sample Size	Arrhythmia Outcome	Arrhythmia Monitoring	No. of Events	Follow-Up	Key Strain Findings
Myocardial infarction								
Ersbøll et al. [56]	2013	Prosp.	988	Composite: VA Appropriate ICD therapy Definite/suspected SCD	Admission with documented VA ICD monitoring in subgroup SCD based on hospital and prehospital records	34	29.7 (IQR: 23.5–32.7) months	GLS and MD were independent predictors of VA. In patients with LVEF < 35%, both GLS and MD were independent predictors of VAs, but only GLS was an independent predictor of VAs in patients with LVEF > 35%.
Haugaa et al. [57]	2013	Prosp.	569	Composite: Sustained VT VF SCD	Not specified	15	30 (IQR: 18) months	GLS was a univariate predictor of VA but not an independent predictor of VA when adjusted for MD. MD was an independent predictor of VA.
Nguyen et al. [63]	2015	Not specified	467	VT	Documented on 24 h ambulatory ECG Monitoring during hospitalization EP study	51	25 (range: 6–43) months	In multivariate analysis, MD was significantly associated with VT, and GLS was borderline significantly associated with VT.
Leong et al. [65]	2015	Retrosp.	206	Appropriate ICD therapy	ICD monitoring	75	24 (IQR: 7.8–24) months	GLS and MD were independently associated with VT.
Haugaa et al. [58]	2010	Prosp.	85	Appropriate ICD therapy	ICD monitoring	38	2.3 (range: 0.6–5.5) years	MD, but not GLS, was an independent predictor of appropriate ICD therapy.
Structural heart disease								
Guerra et al. [66]	2020	Prosp.	203	Any VA detected by ICD	ICD monitoring	74	817 (IQR: 440–1105) days	GLS was an independent predictor of the first VA episode but not recurrent episodes. MD was not associated with VAs.
Heart failure with reduced ejection fraction								
Matsuzoe et al. [70]	2016	Retrosp.	72	Appropriate ICD therapy	ICD monitoring	34	17 (IQR: 0.2–72.5) months	GLS and MD were not independently associated with the VA endpoint. Only LV dyssynergy (SD of peak strain) was independently associated with the VA endpoint.
Hasselberg et al. [68]	2016	Prosp.	170	Composite: VT VF SCA Appropriate ATP Appropriate defibrillator shock therapy	CRT-D monitoring	18	1.9 ± 0.3 years	GLS and MD at baseline were not independent predictors of the VA endpoint. MD at 6 months was an independent predictor of the VA endpoint.
Mornoş et al. [69]	2017	Prosp.	340	Composite: VT VF SCD	Hospital documentationDeath certificate	48	36 ± 9 months	GLS, MD, and the ratio of GLS to MD (GLS/MD) were univariate predictors of VAs, but only GLS/MD was an independent predictor of VAs.
Banasik et al. [130]	2016	Retrosp.	47	Appropriate CRT-D therapy	CRT-D monitoring	29	4 years	MD was greater in patients experiencing VAs. GLS was not reported. No multivariate analyses were performed.
Van der Bijl et al. [131]	2018	Retrosp.	1185	Appropriate CRT-D therapy	CRT-D monitoring	403	55 ± 36 months	No difference in VA events between high vs. low baseline MD but more frequent VA events in those with high MD at 6 months. MD at 6 months was independently associated with VAs. GLS was not reported.
Biering-Sørensen et al. [61]	2017	RCT substudy	1064	Appropriate ICD/CRT-D therapy	ICD/CRT-D monitoring Adjudicated events	254	2.9 (IQR:2.0–3.7) years	GLS and all regional (anterior and inferior) strains were associated with VT/VF, whereas MD was not.
Kutyifa et al. [133].	2013	RCT substudy	1077	VT/VF	ICD/CRT-D monitoring Adjudicated events	- 205 (for baseline associations) - 90 (for associations after 12 months)	2.3 ± 0.9 years	Baseline MD was not associated with VAs. Patients with LBBB who had >15% improvement in MD had a lower risk of VAs.
Biering-Sørensen et al. [72]	2016	Retrosp.	151	Composite: CVD Appropriate ICD therapy	ICD monitoring CVD from the national cause of death registry	40	2.3 (IQR: 1.5–3.1) years	Neither MD nor GLS was associated with VAs.
Non-ischemic dilated cardiomyopathy								
Haugaa et al. [74]	2012	Prosp.	94	Composite: Appropriate ICD therapy Sustained VT Cardiac arrest Cardiac syncope	Not specified	12	22 (Range:1–46) months	Both GLS and MD were independent predictors of the VA endpoint.
Kosiuk et al. [134]	2015	Prosp.	20	Composite: VT VF	Holter, duration not specified ICD monitoring	11	70 ± 40 months	Greater MD in patients with VAs and MD was independently associated with the VA endpoint.
Negishi et al. [76]	2016	Retrosp.	124	Appropriate ICD therapy	ICD monitoring	36	3.8 (IQR: 2.2–6.0) years	GLS but not MD was an independent predictor of VAs.
Melichova et al. [75]	2021	Prosp.	290	Composite: SCD Shock from ICD Sustained VT	Medical records (ICD therapy, ECG, Holter, aborted cardiac arrest) Cause of death registry	32	22 ± 12 months	Both GLS and MD were independent predictors of VA endpoint.
Hypertrophic cardiomyopathy								
Haland et al. [77]	2016	Prosp.	150 HCM	Composite: Sustained and non-sustained VT Previous aborted cardiac arrest	24–48 h Holter monitoring ICD monitoring	37	Not specified	GLS and MD were univariate predictors of the VA endpoint, but only MD was an independent predictor.
Candan et al. [78]	2017	Prosp.	63	Appropriate ICD therapy	ICD monitoring	17	3 years (21.5 ± 6.9 months)	GLS and MD were independent predictors of VAs.
Jalanko et al. [83]	2016	Prosp.	31	Non-sustained VT	24 h Holter monitoring	11	N/A	Both GLS and MD were associated with non-sustained VT in univariate analysis, but only MD was independently associated with non-sustained VT.
Popa-Fotea et al. [81]	2020	Prosp.	47	Non-sustained VT	24 h Holter monitoring	16	N/A	GLS, RV, and LV MD were univariate predictors of non-sustained VT, but only RV and LV MD were independent predictors of non-sustained VT.
Chagas cardiomyopathy								
Barros et al. [84]	2016	Retrosp., case-control study	62	Clinically indicated implantation of ICD.	N/A	28	N/A	MD and GLS were more abnormal in the group with ICD, and both were independent markers of previous events precipitating ICD.
Azevedo et al. [85]	2021	Prosp.	77	Composite: VES Non-sustained VT	24 h Holter	Not specified	N/A	Both GLS and MD were associated with non-sustained VT in univariate analysis, but only MD was independently associated with non-sustained VT, paired VES, and VES in bigeminy.
Long QT syndrome								
Haugaa et al. [132]	2008	Prosp.	73 LQTS 20 healthy individuals	History of either: Documented arrhythmia Syncope Cardiac arrest	N/A	33	Not specified	LQTS patients with a history of arrhythmia had a higher MD than those without arrhythmia. GLS not reported.
Haugaa et al. [86]	2010	Prosp.	101 LQTS 35 healthy individuals	History of either: Documented arrhythmia Syncope Cardiac arrest	N/A	48	N/A	LQTS patients with a history of arrhythmia had a higher MD but similar GLS compared to those without arrhythmia.
Lamin A/C mutation								
Haugaa et al. [87]	2015	Prosp.	33	Composite: Non-sustained VT VT VF	Not specified	11	Not specified	Patients with any ventricular arrhythmia had higher MD but similar GLS compared to those without ventricular arrhythmia.
Arrhythmogenic cardiomyopathy								
Lie et al. [93]	2018	Prosp.	117	VT Cardiac arrest Appropriate ICD shock	ECG Holter monitoring ICD monitoring	18	2.0 (IQR:0.5–3.5) years	Patients with VAs had reduced LV and RV strain and higher LV and RV MD. RV strain and LV MD were independently associated with VAs.
Kirkels et al. [96]	2021	Retrosp.	160	History of either: Sustained VT Appropriate ICD therapy Aborted cardiac arrest	N/A	47	N/A	Patients with a history of VAs had reduced LV GLS and RV strain and higher RV MD than those without VA history. RV MD was independently associated with VAs.
Sarvari et al. [95]	2011	Prosp. Case-control study	42 symptomatic 27 asymptomatic 30 healthy	History of either: VT VF	N/A	42	N/A	Patients with a history of VAs had lower LV and RV strain and higher LV and RV MD. Only RV MD was independently associated with a history of VAs.
Mitral valve prolapse								
Ermakov et al. [97]	2019	Retrosp.	59 MVP	History of: Ventricular couplets Ventricular bigeminy Non-sustained VT VT ICD for aborted cardiac arrest	N/A	32	N/A	MD was higher but similar GLS was seen in patients with a history of VA compared to those without arrhythmia. MD was independently associated with a history of VA.
Brugada syndrome								
Scheirlynck et al. [91]	2020	Prosp.	175 BrS	History of: Sustained VT VF Aborted cardiac arrest	Medical records	19	Not specified	Patients with a history of VAs had higher LV MD but similar LV GLS, RV strain, and RV MD compared to those without VA history. High LV MD was independently associated with VA history.
Elite Athletes								
Lie et al. [92]	2021	Cross-sectional study	43 athletes with VT and 30 healthy athletes	Composite of life-threatening VAs: VF Sustained VT Aborted cardiac arrest Appropriate ICD therapy	24 h Holter monitoring ECG Telemetry ILR monitoring Intracardiac device monitoring	23	N/A	MD was higher and GLS was lower in VA patients. Only MD was independently associated with life-threatening VAs.
Idiopathic ventricular fibrillation								
Groeneveld et al. [99]	2021	Retrosp. Case-control study	47 IVF 47 healthy individuals	VF	N/A	N/A	N/A	IVF patients had lower GLS, higher MD, and higher post-systolic index than matched controls. No adjusted analyses were performed.
Repaired Tetralogy of Fallot								
Van Grootel et al. [89]	2019	Prosp.	151 ToF	Composite: Death HF Reintervention Hospitalization for cardiac reasons Symptomatic ventricular and supraventricular arrhythmias.	Regularly checked at an outpatient clinic	62	71.5 (IQR: 64–75.3) months	GLS, RV strain, and apical rotation were univariate predictors of the composite outcome. Only apical rotation was independently associated with the composite outcome. MD was not investigated.
Unexplained syncope								
Falsing et al. [98]	2021	Retrosp.	288	VT	ILR monitoring	36	2.9 (IQR:1.3–3.5) years	GLS was independently associated with VT. MD was not associated with VT.

Abbreviations: Prosp., prospective; Retrosp., retrospective; HCM, hypertrophic cardiomyopathy; LQTS, long QT syndrome; MVP, mitral valve prolapse; Brs, Brugada syndrome; VT, ventricular tachycardia; IVF, idiopathic ventricular fibrillation; ICD, implantable cardioverter-defibrillator; SCD, sudden cardiac death; VF, ventricular fibrillation; SCA, sudden cardiac arrest; CRT, cardiac resynchronization therapy; VES, ventricular extrasystoles; HF, heart failure; VA, ventricular arrhythmia; ECG, electrocardiogram; GLS, global longitudinal strain; MD, mechanical dispersion; LVEF, left ventricular ejection fraction; LV, left ventricle; RVMD, right ventricular mechanical dispersion; RV, right ventricle; MD, mechanical dispersion; LBBB, left bundle branch block.

## 6. Future Directions

### 6.1. An Unexplored World of Strain Measures

Even though GLS, MD, and regional strain have been the most widely studied strain measures in terms of predicting VAs, other potential strain measures of myocardial mechanics could be of value. These include measures of paradoxical motion and myocardial work. To date, however, their associations with VAs have only been sparsely investigated.

**Figure 3:**
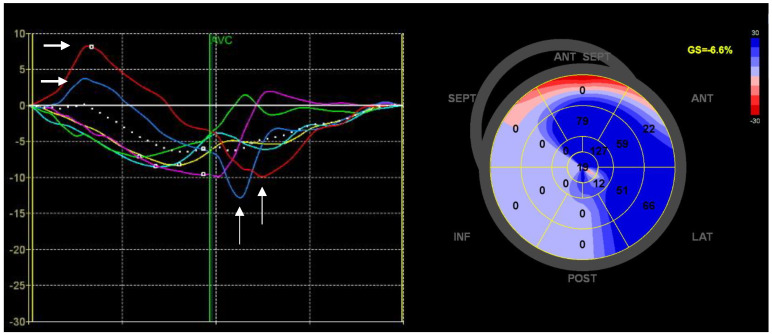
Paradoxical motion. The figure shows an example of strain analysis for quantifying paradoxical motion in a patient with ischemic cardiomyopathy and ICD who experienced appropriate therapy after implantation. On the left side, segmental strain curves from the apical 4–chamber view are shown, with arrows illustrating early systolic lengthening and post-systolic shortening in the lateral wall segments (blue and red strain curves). On the right side, a bulls–eye plot of the post-systolic index in all segments is presented, also showing a high post-systolic index in the anterior, lateral, and anteroseptal segments.

In certain settings, the motion of myocardial tissue is altered such that segments of the LV may lengthen when they are supposed to shorten. Such a feature is termed paradoxical motion and is usually expressed as either early systolic lengthening or post-systolic shortening [135]. Figure 3 shows examples of paradoxical motion in a patient with ischemic cardiomyopathy and ICD who experienced appropriate therapy after implantation. These abnormal movements have been linked to ischemic heart disease; they often co-exist, and their presence has been thought to indicate ischemic segments with potential tissue viability [136,137]. However, they can also develop in other settings, including conduction abnormalities and mitral annular disjunction [138,139]. It should also be noted that, to some extent, they may appear under normal circumstances as part of normal physiology [140,141]. Given that these features of myocardial deformation seem to be closely linked to ischemia and the extent of myocardial affliction, they could represent markers of elevated VA risk. Indeed, in the previously mentioned multicenter study by Haugaa et al. (n: 569, VA events: 15), the authors found that patients who developed VAs had a higher degree of post-systolic shortening, expressed as the post-systolic strain index (PSSI), than patients who did not develop VAs. This was also evident in those with LVEF > 35%. By extension, PSSI was also a univariate predictor of VAs, yielding similar predictive performance by C-statistics as MD, but was not an independent predictor of VAs in multivariate adjustments with MD [57]. Similarly, Groeneveld et al. have also shown that patients with idiopathic VF exhibit more frequent and widespread post-systolic shortening when compared to age- and sex-matched healthy controls [99].

**Figure 4:**
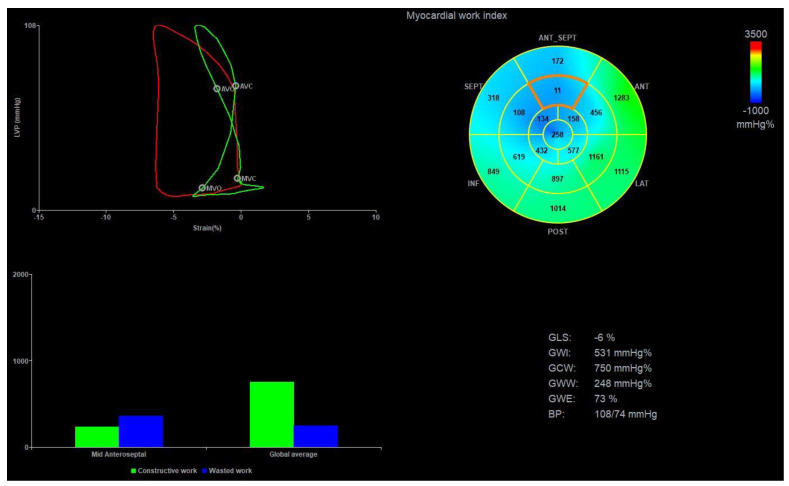
Pressure–strain loop analysis. The figure shows an example of pressure–strain loop analysis for quantifying myocardial work in a patient with ischemic cardiomyopathy and ICD who experienced appropriate therapy after implantation. The upper left–sided panel shows the pressure–strain loop, the area of which corresponds to the myocardial work index. The red profile shows the global myocardial work, showing normal clockwise looping but overall reduced myocardial work. The green profile depicts the mid–anteroseptal segment, showing a counterclockwise looping. The bottom left panel shows the relative extent of constructive work (green bars) and wasted work (blue bars), illustrating a fairly high amount of wasted work, particularly in the mid–anteroseptal segment (bars on the left). The top right panel is a bulls–eye plot showing all regional myocardial work estimates, and the global values for all work measures are shown in the bottom right panel, along with blood pressure and global longitudinal strain.

The most recent advancement in strain imaging has been the development of a non-invasive method for estimating myocardial work through pressure–strain loop analysis. This method was introduced by Russell et al. in 2012 and allows for the estimation of myocardial work by combining information on the strain, cuff arterial blood pressure, and valvular event timing [142,143]. Figure 4 shows an example of myocardial work in a patient with ischemic cardiomyopathy with ICD and appropriate therapy for VA. Similar to other strain measures, the reproducibility has been reported to be excellent [142,144,145,146]. Since this technique incorporates blood pressure as a surrogate of afterload, it may provide a more valid assessment of systolic function. Expert statements have since been published to guide the practical approach to measuring myocardial work [147]. In addition, normal values and the impact of age and sex have been detailed in several studies, including a meta-analysis [144,148,149,150,151]. Aside from quantifying myocardial work, the method also allows for the quantification of wasted work, constructive work, and work efficiency [152], all of which are influenced by the presence of paradoxical motion. Accordingly, these metrics may be used as a means to quantify paradoxical motion and thereby indicate the risk of VAs. Myocardial work measures have shown potential value in a wide range of settings and potentially provide clinical information superior to GLS [145,153,154]. A case report by Jaworski et al. alluded to the potential application of regional work in a patient who developed VF [155]. Furthermore, in a study of 110 patients with hypertrophic cardiomyopathy, Hiemstra et al. reported that constructive work was a viable predictor of a combined clinical endpoint, which included aborted sudden cardiac death and appropriate ICD therapy, which constituted 11 of the observed 24 events [146]. However, no separate details were reported about the association between myocardial work measures and VA endpoints. Accordingly, the potential use of myocardial work in the context of VAs remains to be fully elucidated.

### 6.2. Extending Current Findings into Clinical Practice

As outlined throughout this review, several studies have shown associations between strain measures and VAs across a broad range of cardiovascular disease groups, highlighting their potential clinical value. However, no study has yet to evaluate whether any of these strain measures may actually be used to guide clinical management, i.e., by randomizing patients to ICD based on a strain measure. While studies are still needed to explore whether measures of paradoxical motion or myocardial work relate to VAs, trials are needed to examine whether the observed associations between VAs and strain measures such as GLS or MD would translate into clinical benefit if they were to be applied for selecting ICD candidates.

## 7. Conclusions

Speckle-tracking echocardiography allows for a comprehensive quantification of the intricate myocardial mechanics that develop in various cardiac disorders. Abnormalities in several strain measures, including regional strain, GLS, and MD, have been shown to be associated with an increased risk of VAs, whereas strain measures of paradoxical motion and myocardial work still need to be explored further in this context. GLS and MD have been most widely investigated and may provide important information for assessing the risk of VAs in several settings, even in patients with LVEF > 35%. Consequently, they could be useful for identifying patients who could stand to benefit from an ICD for the prevention of sudden cardiac death. However, trials are needed to substantiate this further.

## Figures and Tables

**Table 1:** Advantages and limitations of strain.

Strengths
On the verge of guideline implementation
Direct tissue measure
Can investigate each fiber aspect
High reproducibility, automatic options
Angle independent (compared to Doppler)
Can provide regional details
Can provide measures of diastolic function and dyssynchrony
Changes typically precede changes in LVEF
**Limitations**
Vendor dependency Loading dependency

## Data Availability

Data sharing not applicable.
